# Graphite as a Long‐Life Ca^2+^‐Intercalation Anode and its Implementation for Rocking‐Chair Type Calcium‐Ion Batteries

**DOI:** 10.1002/advs.201902129

**Published:** 2019-10-16

**Authors:** S. J. Richard Prabakar, Amol Bhairuba Ikhe, Woon Bae Park, Kee‐Choo Chung, Hwangseo Park, Ki‐Jeong Kim, Docheon Ahn, Joon Seop Kwak, Kee‐Sun Sohn, Myoungho Pyo

**Affiliations:** ^1^ Department of Printed Electronics Engineering Sunchon National University Suncheon Chonnam 57922 Republic of Korea; ^2^ Faculty of Nanotechnology and Advanced Materials Engineering Sejong University Seoul 05006 Republic of Korea; ^3^ Department of Bioscience and Biotechnology Sejong University 209 Neungdong‐ro, Kwangjin‐gu Seoul 143‐747 Republic of Korea; ^4^ Beamline Division Pohang Accelerator Laboratory (PAL) Pohang 37673 Republic of Korea

**Keywords:** Ca‐ion batteries, calcium intercalation, cointercalation, graphite anodes, organic cathodes

## Abstract

Herein, graphite is proposed as a reliable Ca^2+^‐intercalation anode in tetraglyme (G_4_). When charged (reduced), graphite accommodates solvated Ca^2+^‐ions (Ca‐G_4_) and delivers a reversible capacity of 62 mAh g^−1^ that signifies the formation of a ternary intercalation compound, Ca‐G_4_·C_72_. Mass/volume changes during Ca‐G_4_ intercalation and the evolution of in operando X‐ray diffraction studies both suggest that Ca‐G_4_ intercalation results in the formation of an intermediate phase between stage‐III and stage‐II with a gallery height of 11.41 Å. Density functional theory calculations also reveal that the most stable conformation of Ca‐G_4_ has a planar structure with Ca^2+^ surrounded by G_4_, which eventually forms a double stack that aligns with graphene layers after intercalation. Despite large dimensional changes during charge/discharge (C/D), both rate performance and cyclic stability are excellent. Graphite retains a substantial capacity at high C/D rates (e.g., 47 mAh g^−1^ at 1.0 A g^−1^ s vs 62 mAh g^−1^ at 0.05 A g^−1^) and shows no capacity decay during as many as 2000 C/D cycles. As the first Ca^2+^‐shuttling calcium‐ion batteries with a graphite anode, a full‐cell is constructed by coupling with an organic cathode and its electrochemical performance is presented.

## Graphical abstract

Graphite can intercalate/deintercalate Ca^2+^ ions with excellent cyclability and rate capability. Calcium ion batteries, which consist of a graphite anode and a perylene‐3,4,9,10‐tetracarboxylic dianhydride cathode in Ca(TFSI)_2_/G_4_, can operate in a rocking‐chair mode and deliver a reversible capacity of ≈80 mAh g^−1^ with good stability.

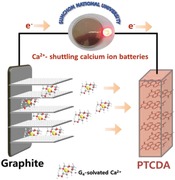

## 1 Introduction

In the quest for the next generation rechargeable batteries, divalent‐ion batteries (Mg^2+^ and Ca^2+^) that could operate under the same principles as the current market leaders, lithium‐ion batteries (LIBs), would be advantageous in several aspects.^1^ Among them, the utmost merit would be that divalent ions are less prone to a dendritic growth during plating, which increases the chances for the implementation of metallic anodes and the fabrication of high‐capacity batteries (2205 and 1337 mAh g^−1^ for Mg and Ca, respectively). Although there are some report on the dendrite growth in a divalent system,^2^ the possible absence of dendrites also can significantly reduce the level of safety concerns, which would simplify both the cell configuration and the ancillary circuits.

On this ground, magnesium‐ion batteries (MIBs) were first selected as promising candidates for divalent‐ion batteries, and these have been extensively studied during the past two decades.^3^, ^4^ Besides the sluggish Mg^2+^‐transport within a cathode matrix due to the high charge density of Mg^2+^,^5^ however, Mg^2+^‐impermeability through passivation layers on a Mg surface posed difficulties in using common organic electrolytes.^6^, ^7^ Therefore, though there were few reports on an artificial Mg^2+^‐conductive interface on a Mg anode surface,^8^ most research on MIBs have been devoted to the design of new electrolyte systems to solve this problem.^9^, ^10^, ^11^, ^12^, ^13^, ^14^, ^15^, ^16^, ^17^, ^18^, ^19^, ^20^, ^21^, ^22^, ^23^ As a result, significant improvements have been made in electrolytes compatible with a Mg metallic anode, but the commercial viability of MIBs is still far from complete due to an insufficient anticorrosive nature and/or to a relatively low level of anodic stability in these electrolytes.^22^, ^23^, ^24^, ^25^, ^26^, ^27^

Compared with MIBs, calcium‐ion batteries (CIBs) would be a more attractive option for divalent‐ion batteries, because higher cell potentials (standard reduction potentials of −2.87 and −2.37 V for Ca/Ca^2+^ and Mg/Mg^2+^, respectively) and faster reaction kinetics (charge densities of 0.49 vs 1.28 e Å^−3^ for Ca^2+^ and Mg^2+^, respectively) can be expected.^28^ CIBs, however, also suffer from irreversible calcium plating/stripping on metallic anodes at room temperature (RT). Previous research revealed that, although the dissolution of Ca^2+^ from a metallic calcium is possible to some extent at RT,^29^ calcium plating either on calcium or on a noble metal is almost completely hampered due to a buildup of surface film.^30^, ^31^ This problem could be partially resolved by increasing the operation temperature (100 °C) and limiting the ion‐pairing of electrolytes for an easier migration of Ca^2+^ through the surface film.^32^ However, non‐negligible over‐potentials (0.5–0.9 V) for calcium plating even at high temperature indicated insufficient adaptability to practical CIBs operable at RT. Recent research has also pointed out that CaH_2_, which is spontaneously formed in Ca(BH_4_)_2_/tetrahydrofuran during charge/discharge (C/D), can alleviate the surface passivation, but the slow formation of CaH_2_ and the low anodic stability of the electrolyte (≈3 V) requires further improvement.^33^

Because of a lack of reliable calcium plating/stripping systems, plausible anode materials or Ca as negative electrode, were coupled with Ca^2+^ insertion/deinsertion cathodes, and their possible use as CIB anodes was indirectly evaluated with very less or no detailed electrochemical studies for an anode alone.^34^, ^35^, ^36^, ^37^, ^38^, ^39^, ^40^, ^41^ For example, Sn foil was implemented as a calcium‐alloying type anode along with a graphite cathode.^36^ A full‐cell operated reasonably well in a dual‐ion mode, but the electrochemical properties of Sn alone and its contribution to full‐cell performance were unclear. There has also been a report on dual‐graphite CIBs, which claimed Ca^2+^‐intercalation into a graphite anode in carbonate solvents. Likewise, no detailed information on Ca^2+^‐intercalation behaviors has been provided, which suggests that the possibility of the surface adsorption of Ca^2+^ in carbonate solvents could not be eliminated (We could not identify reversible Ca^2+^‐intercalation/deintercalation in the same electrolyte system, see the Results and Discussion Section).^40^ Intercalation of Ca^2+^ into natural graphite and HOPG was also demonstrated in a Ca(TFSI)_2_/dimethyl sulfoxide (DMSO) electrolyte with a possiblity of solvent (DMSO) cointercalation. However, this demonstration also lacks detailed electrochemical analysis.^41^ The anode materials used in CIBs to this point are summarized in **Table** **1**.

**Table 1:** Anode materials and their electrochemical properties investigated for CIBs till now

Anode	Electrolyte	Reversible capacity	Final capacity/no. of cycles	Remarks	Ref.
Ca metal	Ca(BF_4_)_2_/carbonates	21 mC	15 mC/30 cycles	Results measured at 100 °C	32
Ca metal	Ca(TFSI)_2_/Monoglyme	80 mAh g^−1^	only a few cycles	Full‐cell with α‐MoO_3_	34
Li metal	LiPF_6_/Ca(PF_6_)_2_/EC+EMC+PC+DMC	95 mAh g^−1^	75 mAh g^−1^/1500 cycles	Calcium‐ion based tri‐ion battery (CTIB) with graphite	35
Au (substrate)	Ca(BH_4_)_2_/THF	1 mAh cm^−2^	N/A	3‐electrode system	33
Sn foil	Ca(PF_6_)_2_/carbonates	(theoretical) 530 mAh g^−1^	N/A	Dual‐ion cell with graphite	36
Sn foil	Ca(PF_6_)_2_ EC/PC/DMC/EMC	86 mAh g^−1^	70 mAh g^−1^/1000 cycles	Dual ion hybrid battery with AC	39
V_2_O_5_ (crystalline)	Ca(ClO_4_)_2_/acetonitrile	N/A	N/A	Full‐cell with CaCo_2_O_4_	29
Graphite (MCMB)	Ca(PF_6_)_2_/carbonates	N/A	N/A	Dual‐ion cell with graphite	40
Graphite (KS6L)	Ca(TFSI)_2_/tetraglyme	62 mAh g^−1^	51 mAh g^−1^/2000 cycles	This work	

Graphite is one of the most reliable anode materials, and is predominantly used in commercialized LIBs.^42^ Graphite can also accommodate K^+^ ions through a staging process (KC_8_).^43^, ^44^ However, the smaller binding energy (i.e., less stabilization) between Na^+^ ions and graphene layers renders the formation of Na^+^‐intercalated compounds difficult (NaC_64_).^45^ This implies that the increase in strain energies via the interlayer expansion of graphite is not a crucial factor in determining intercalability, because the ionic radius of K^+^ (1.38 Å) is substantially larger than that of Na^+^ (1.02 Å). In fact, Na^+^ ions are known to reversibly cointercalate with diglyme, despite a significant increase in the interlayer spacing (3.35–11.62 Å).^46^, ^47^ When fully charged, a stage‐I compound, Na(diglyme)_2_C_20_, forms, and delivers a capacity of ≈100 mAh g^−1^. The possibility of a cointercalation of Mg^2+^ and diglyme into graphite has also been claimed. However, intercalation has been limited to a stage‐VI compound with the formation of disordered graphitic domains.^48^

Herein, we describe the cointercalation of Ca^2+^ with tetraglyme (G_4_) into graphite. In contrast to the irreversibility observed in other carbonate‐ or ether‐type solvents, graphite can reversibly accommodate Ca^2+^ in G_4_. Excellent reversibility is maintained for as many as 2000 C/D cycles with no degradation. We show the stepwise intercalation of Ca^2+^ via various analytical and computation tools. The viability of graphite as an anode in a Ca^2+^‐shuttling CIB full‐cell is also presented.

## 2 Results and Discussion

First, the possibility of Ca^2+^‐intercalation into graphite was examined in carbonate‐based electrolytes containing calcium bis(trifluoromethanesulfonyl)imide (Ca(TFSI)_2_). A coin cell with a configuration of graphite│electrolyte│activated carbon (AC) was fabricated and its electroactivity was tested via cyclic voltammetry. An AC electrode was used as the counter electrode instead of Ca metal, to avoid the complexity ascribed to the Ca surface passivation in the electrolytes used. An excess amount of AC relative to graphite was used to minimize a potential shift in AC during C/D. Based on the capacitance value of AC (≈150 F g^−1^, Figure S1A, Supporting Information), an AC mass that was ≈23‐fold greater than the graphite mass (typically 45 vs 2 mg) was loaded to maintain the potential of AC during C/D within ±0.05 V, even when the graphite delivered a Ca^2+^‐storage capacity of 100 mAh g^−1^. Note that the excess amount of AC in this study means the great mass loading of AC to minimize potential variations of an AC electrode during C/D. The potential of an AC electrode in Ca(TFSI)_2_ was also calibrated by using polyvinyl ferrocene (PVFc) as an internal standard, which was immobilized on a Pt substrate. A voltammogram was recorded versus AC, and the half‐wave potential (E_1/2_) of PVFc (+0.24 V vs AC) was ascertained (Figure S1B, Supporting Information). The reference potential of an AC electrode in 1.0 m Ca(TFSI)_2_, therefore, was determined to be +0.30 V versus standard hydrogen electrode (SHE). Hereafter, all the electrochemical potentials will be stated relative to SHE, unless otherwise noted.

**Figure 1:**
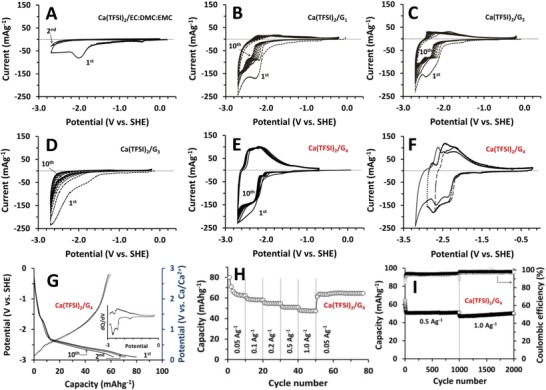
Cyclic voltammograms of graphite in A) EC:DMC:EMC (4:3:2), B) G_1_, C) G_2_, D) G_3_, and E) G_4_ solutions containing 1.0 m Ca(TFSI)_2_ at a scan rate of 0.5 mV s^−1^. F) Cyclic voltammograms of graphite in Ca(TFSI)_2_/G_4_ with different *E* ^low^. G) Representative C/D profiles (0.05 A g^−1^), H) rate performance, and I) cyclic stability of graphite in Ca(TFSI)_2_/G_4_ when cycled between −0.3 and −2.9 V. Inset in (G) shows a *d*Q/*d*V curve corresponding to the 10th C/D profile.

When cycled in carbonate solvents containing 1.0 m Ca(TFSI)_2_, graphite (flake‐type KS6L, TIMCAL) showed no signs of reversible Ca^2+^ intercalation/deintercalation (**Figure** **1**A). The cathodic process observed during the 1st negative scan in EC:DMC:EMC (ethylene carbonate:dimethyl carbonate:ethyl methyl carbonate, 4:3:2, v/v/v) was followed by no anodic currents during the 1st positive scan. Subsequent C/D also showed negligible levels of current. Identical behaviors were observed in other carbonate solutions (Figure S2, Supporting Information), which signified that the 1st cathodic process rendered graphite completely electroinactive. It was believed that a surface film formed on the graphite in the carbonate‐based electrolytes during the 1st negative scan impeded Ca^2+^‐transport, though dual‐ion CIBs composed of graphite│Ca(PF_6_)_2_/EC:DMC:EMC│graphite had been reported.^40^ In fact, a substantial magnitude of impedance was obvious after the 1st discharge, which clearly suggested the surface passivation in the carbonate solvents (Figure S3, Supporting Information).

The failure to obtain a reversible intercalation behavior in carbonate‐type electrolytes urged us to shift our attention to glyme‐based electroytes, which are known to effectively solvate various cations including Mg^2+^.^49^, ^50^, ^51^ Since a degree of solvation is affected by the chain length of glymes, monoglyme (G_1_), diglyme (G_2_), triglyme (G_3_), and G_4_ were chosen to investigate the relationship between the solvation and the Ca^2+^ intercalation. The voltammetric behaviors of graphite were strikingly different with the chain length of glymes. The particular trend, however, could not be established, since a slight change in the chain length can significantly affect the stability of a solvation sheath.^50^ The voltammograms in G_1_, G_2_, G_3_, and G_4_ showed conspicuous cathodic currents beginning at −2.2 V during the 1st negative scan (Figure 1B–E). However, the following positive scan and continuous cycles showed different current behaviors with a type of glyme. A negligible level of anodic currents and a gradual decrease in the cathodic currents with cycling in G_1_, G_2_, and G_3_ (Figure 1B–D, respectively), were in contrast to the reversible and stable redox processes in G_4_ (Figure 1E). Since Ca(TFSI)_2_/glymes were electrochemically inert within the potential window examined (Figure S4, Supporting Information), the significant cathodic currents in glymes were believed to be associated with graphite reduction and with a concomitant Ca^2+^‐intercalation. On the other hand, different degrees of the reversibility with different types of glyme appeared to imply different degrees of solvation.^49^ The weak levels of solvation in G_1_, G_2_, and G_3_ seemed to result in bare Ca^2+^‐intercalation (i.e., no solvent cointercalation) and irreversible entrapment of Ca^2+^, which led to slight (or negligible) anodic currents for the 1st positive scan and to a gradual decrease in the cathodic currents during following cycles. In contrast, the strong solvation of Ca^2+^ in G_4_ likely resulted in cointercalation of G_4_, which alleviated the electrostatic interactions and secured the reversible intercalation/deintercalation of Ca^2+^ (see below for the validation of this notion).

A reversible cyclic voltammogram of graphite in Ca(TFSI)_2_/G_4_ clearly suggested that graphite can be used as an anode in CIBs. Note that the reversible electrochemistry in Ca(TFSI)_2_/G_4_ was also the case for sphere‐shaped graphite (mesocarbon microbeads, MCMB, Figure S5, Supporting Information). To determine the low potential‐cutoff (*E* ^low^) with no calcium plating, the *E* ^low^ was stepwise lowered from −2.7 to −2.9 and to −3.2 V (Figure 1F). A comparison of steady‐state voltammograms revealed a Coulombic efficiency that was suddenly decreased from ≈95% to 60% when the *E* ^low^ was decreased from −2.9 to −3.2 V, which indicated the possibility for irreversible calcium plating at −3.2 V. The charge (reduction, Ca^2+^ intercalation) and discharge (oxidation, Ca^2+^ deintercalation) capacities when *E* ^low^ = −2.9 V were 64 and 61 mAh g^−1^, respectively.

While limiting the *E* ^low^ to −2.9 V, galvanostatic C/D was performed at 0.05 A g^−1^ (Figure 1G). The representative C/D profiles showed that, while the discharge capacities were almost invariant at 60 mAh g^−1^, the initial charge capacity of 80 mAh g^−1^ was quickly converged to 62 mAh g^−1^ within 10 cycles. Assuming no further electrolyte decomposition, a capacity of 62 mAh g^−1^ corresponded to the formation of Ca·C_72_. Note that a *d*Q/*d*V curve of the 10th C/D (Figure 1G, inset) was identical to the cyclic voltammogram shown in Figure 1F. In contrast to capacity of ≈100 mAh g^−1^ obtained at the fully charged stage‐1 Na(diglyme)_2_C_20_ compound,^46^ a capacity of 62 mAh g^−1^ in the current work which corresponded to the formation of Ca·C_72_ compound may suggest a stage number higher than 1. The lower capacity of The Ca^2+^ intercalation/deintercalation was not severely affected by C/D rates (Figure 1H). A charge capacity of 47 mAh g^−1^ was delivered at 1.0 A g^−1^, retaining ≈76% of the reversible capacity obtained at 0.05 mA g^−1^ (62 mAh g^−1^). On returning the C/D rate to 0.05 A g^−1^, the capacity was slightly increased to 63–64 mAh g^−1^. The cyclic stability was also surprisingly excellent (Figure 1I). Once stabilized, graphite experienced no further capacity fading during 2000 C/D cycles in Ca(TFSI)_2_/G_4_. It rather showed a slight increase in capacities with cycling.

**Figure 2:**
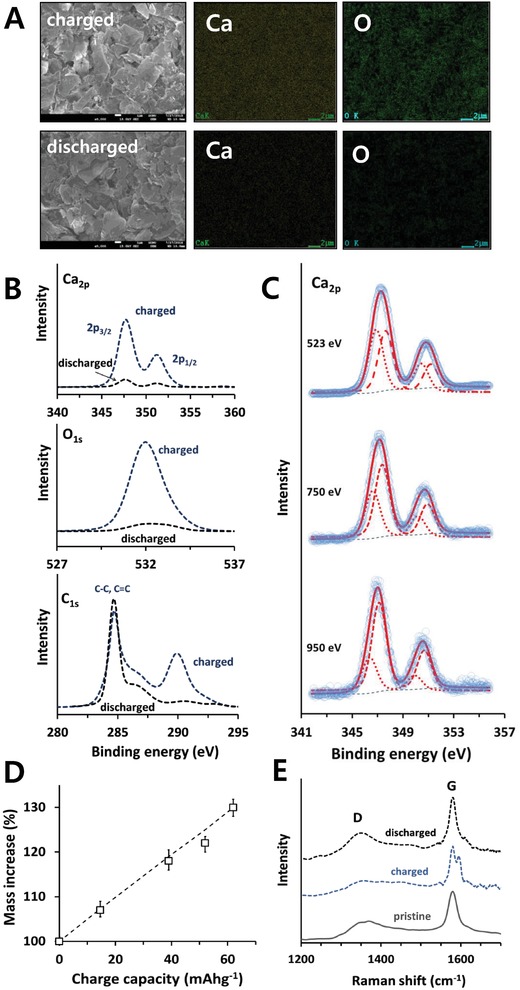
A) Field emission scanning electron microscopy and EDX images and B) XPS spectra of charged and discharged graphites at −2.9 and −0.2 V versus SHE, respectively. C) Synchrotron XPS spectra of charged graphite with different photon energies. Circle: experimental data; dotted/dashed lines: fitting curves; solid line: sum of fitting curves. D) Mass increase of graphite with a charge. Dashed line = theoretical curve based on Ca‐G_4_ intercalation. E) Raman spectra of pristine, charged, and discharged graphites.

In addition to electrochemical verifications, reversible Ca^2+^ intercalation/deintercalation was also confirmed by comparing the calcium content between charged and discharged samples. As expected, energy dispersive X‐ray (EDX) images (**Figure** **2**A) and the X‐ray photoelectron spectroscopy (XPS) spectra (Figure 2B) indicated a high concentration of calcium in charged graphite in contrast to the relatively small content in discharged graphite. EDX and XPS investigations also showed a concomitant increase/decrease in the oxygen content, which was interesting because the synchronous changes in oxygen and calcium content implied a reversible intercalation/deintercalation of G_4_ with Ca^2+^. In fact, the atomic ratios of O/Ca, calculated from the EDX and XPS spectra were 4.75 and 4.62 for a charged sample, respectively, which indicated the intercalation of Ca^2+^ solvated by a single G_4_ molecule (Ca‐G_4_). The XPS spectra of C_1s_ also supported a reversible intercalation/deintercalation of Ca‐G_4_. While the peak intensity at 290.0 eV was negligible in discharged graphite, a charged sample showed an intense peak that was believed to be due to the ethereal carbon of G_4_.^52^ It is worth mentioning here that the XPS spectrum of discharged graphite that was cycled in Ca(TFSI)_2_/G_2_ showed an intense calcium peak, in contrast to that cycled in Ca(TFSI)_2_/G_4_, due to Ca^2+^‐entrapment (Figure S6, Supporting Information).

The slight content of Ca^2+^ in a discharged sample cycled in Ca(TFSI)_2_/G_4_ (Figure 2B) was not ascribed to entrapped Ca^2+^, but was due to the Ca^2+^ that existed on the graphite surface (via surface adsorption and/or solid‐electrolyte‐interface). Synchrotron XPS, which is more surface‐sensitive than conventional XPS, revealed the presence of surface Ca^2+^ (Figure 2C). The Ca_2p3/2_ peaks were well‐fitted by two Voigt functions centered at 346.6 and 347.5 eV, which were considered to originate from surface Ca^2+^ and intercalated Ca‐G_4_, respectively. Different chemical environments for the two different Ca^2+^ ions (surface adsorbed and intercalated) cause their binding energies to differ. As the sampling depth was increased by changing the excitation energies from 523 to 750 and 950 eV, a gradual decrease in the relative intensities of the low binding‐energy peak (346.6 eV) was evident, which indicated that the calcium peaks for a discharged sample in Figure 2B were correlated with the surface Ca^2+^.

A reversible capacity of 62 mAh g^−1^ and a cointercalation of G_4_ indicated that a full charge of graphite at −2.9 V could produce a ternary intercalation compound, Ca‐G_4_·C_72_, and increase the mass of graphite by 30%. The variation in graphite masses indeed showed a linear increase with depth‐of‐charge (Figure 2D). The mass was changed in a linear fashion, and almost traced a hypothetical line that was based on the assumption of a mass increase by intercalation of Ca‐G_4_.

We further characterized the intercalation behavior of charged and discharged graphites by ex situ Raman spectroscopy (Figure 2E). While the D band was weak and almost invariant, the change in the G band was conspicuous with charge and discharge.^53^, ^54^ When charged, the intense G band peak of pristine graphite (1580 cm^−1^) was split into two peaks centered at 1580 and 1596 cm^−1^, which are ascribed to the symmetry change at the boundary layers and/or the electronic effect of the intercalants.^55^, ^56^ After discharge, the two peaks were merged to show a single peak as in pristine graphite. The doublet G band in charged graphite was interesting because it is frequently observed in graphite‐intercalation‐compounds when a stage number is equal to or higher than 2,^56^ which implied that Ca‐G_4_·C_72_ would have a high stage number. The intensity ratio between the D and G bands (*I*_D_/*I*_G_) also indicated the disorder induced by Ca‐G_4_ intercalation. The *I*_D_/*I*_G_ of 0.35 in pristine graphite was decreased to 0.28 in a charged sample and increased again to 0.34 in a discharged sample, which were associated with reversible Ca‐G_4_ intercalation/deintercation.

**Figure 3:**
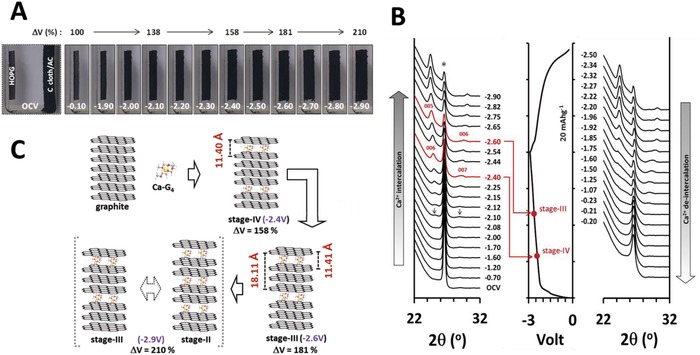
A) Photo images of HOPG showing the volume expansion due to Ca‐G_4_ intercalation during a charge. B) In situ X‐ray diffractograms during C/D and corresponding voltage profiles. C) Schematic illustration of various staging processes for Ca‐G_4_ intercalation into graphite.

Since the cointercalation of G_4_ was expected to induce a significant expansion of the interlayer spacing of graphite, we monitored the volume change of a highly oriented pyrolytic graphite (HOPG) during Ca‐G_4_ intercalation. The HOPG was fixed inside a rectangular glass tube with its basal plane facing an AC electrode (**Figure** **3**A). The tube was filled with Ca(TFSI)_2_/G_4_ and was charged to −2.90 V at a rate of 0.05 A g^−1^. The expansion of the HOPG was monitored in real time using a digital camera (Figure 3A and Video S1, Supporting Information). Upon decreasing the potential, the HOPG began to swell and continuously expand along the crystallographic c‐axis with no noticeable change in the ab‐planes. The HOPG volumes increased to 138 (−2.10 V), 158 (−2.40 V), 181 (−2.60 V), and finally 210% (−2.90 V). Since the volume increase would be limited to 160% even for a stage‐I intercalation compound when Ca^2+^ alone is intercalated (increase from 3.35 to 5.35 Å of an interlayer distance, based on the ionic size of Ca^2+^), such a large volume expansion cannot be explained without considering G_4_ cointercalation.

To understand the cointercalation mechanism and the structural change of graphite, the evolution of synchrotron XRD patterns was examined *in operando* (Figure 3B). It was obvious that the electrochemical intercalation/deintercalation of Ca‐G_4_ induced continuous shifts in the characteristic XRD peaks. The intensity of the (002) peak of graphite at 26.5° (indicated by an asterisk) began to decrease with a charge, and two new peaks appeared at 24.8° and 28.5° from −2.10 V (indicated by arrows). These two peaks continuously moved to lower and higher 2θ with further charging and the intensities became stronger. On the completion of a charge process at −2.90 V, the two peaks were located at 24.4° and 30.0°. Though XRD showed no biphasic patterns, the staging process could be confirmed by assigning the new peaks to (00*n*) and (00*n*+1).^57^ The values of *n* and *I*_c_ (c lattice parameter of a graphite‐intercalation‐compound) were determined using Equations (1) and (2)

(1) $$n\;=\frac{1}{\left[\frac{\sin\theta_{00n+1}}{\sin\theta_{00n}}-1\right]}$$

(2) $$d_{00n}=\frac{I_{c}}{n}\;\;\mathrm{and}\;\;d_{00n+1}=\frac{I_{c}}{n+1}$$

In Equations (1) and (2), *d*_00_ *_n_* and *d*_00_ *_n_*_+1_ are the d‐spacing values of (00*n*) and (00*n*+1) planes, respectively. The calculation results revealed that the integer values of *n* can be obtained only at −2.40 and −2.60 V. For example, 2θ_00n_ of 24.55° and 2θ_00_ *_n_*_+1_ of 29.56° (*d*_00_ *_n_* = 3.622 Å and *d*_00_ *_n_*_+1_ = 3.018 Å) at −2.60 V resulted in *n* of 5.00 and *I*_c_ of 18.11 Å. Since Δ*V* at this potential was 181% (Figure 3A), the possible stage number was determined as “III” with a gallery height of 11.41 Å. The possibility for the formation of other stage compounds was excluded, based on the volume expansion (Figure S7, Supporting Information). Likewise, the stage number of “IV” and the same gallery height of 11.40 Å were obtained at −2.40 V (Δ*V* = 158%). For a fully charged state (−2.90 V), on the other hand, an *n*‐value of 4.4 was obtained, which indicated that the graphite is in the intermediate state in the course of a monophasic transition from stage‐III to stage‐II (see Figure 3C). The HOPG volume expansion of 210% was also less than 220%, which is the expected Δ*V* for stage‐II ((11.41 + 3.35)/6.70 = 2.20). A reversal of the scan direction showed the opposite behaviors in the peak positions and intensities. When fully discharged, the peaks ascribed to the Ca‐G_4_ intercalation completely disappeared and only the (002) peak of pristine graphite remained, which signified good reversibility for intercalation/deintercalation of G_4_‐solvated Ca^2+^ with no entrapment.

A (002) peak at 26.5° was apparent in all XRD patterns. This peak, however, did not originate from the graphite anode, but from the AC that was used as the counter electrode (Figure S8A, Supporting Information). The AC electrode was not punched for in situ XRD measurements, because punching a hole substantially reduces the amount of AC and results in a significant voltage‐drift of the AC electrode during *in operando* XRD measurements. In fact, ex situ‐XRD patterns of the graphite in a fully charged state (i.e., no interference from an AC counter electrode) displayed a negligible (002) peak with strong (00*n*) and (00*n*+1) peaks (Figure S8B, Supporting Information).

We also performed density functional theory (DFT) calculations to investigate the molecular arrangement of Ca‐G_4_ between graphene layers. DFT calculations were executed via three steps. First, solvation energy was calculated for three different Ca‐G_4_ configurations. Second, based on the most stable Ca‐G_4_ configuration, we compared the relative stability of Ca^2+^ ions in G_2_ and G_4_. Finally, using the stable Ca‐G_4_ complex, the most plausible arrangement of Ca‐G_4_ between graphene layers was determined. The first two steps were based on Gaussian DFT calculation. The solvation energy was evaluated from the difference between the total energy of the Ca‐G_4_ complex (*E*_Ca‐G4_) and the sum of the energies of an isolated G_4_ molecule (*E*_G4_) and a Ca atom (*E*_Ca_), as shown in Equation (3) below

(3) $$\mathrm{Solvation}\;\mathrm{energy}=E_{\mathrm{Ca}-G4}-\left(E_{G4}+E_{\mathrm{Ca}}\right)$$

A comparison of the solvation energies for three different Ca‐G_4_ configurations revealed that Ca^2+^ surrounded by a G_4_ molecule is more stable than the other two structures with an extended G_4_ molecule (Figure S9, Supporting Information). Ca^2+^ also proved to be more preferentially associated with G_4_ than with G_2_. The solvation energy of Ca‐G_4_ was substantially lower than that of Ca‐G_2_, which disclosed the reason why the cointercalation occurs only in G_4_. The optimized Ca‐G_4_ and Ca‐G_2_ structures and their corresponding solvation energy values are shown in Figure S10 (Supporting Information).

**Figure 4:**
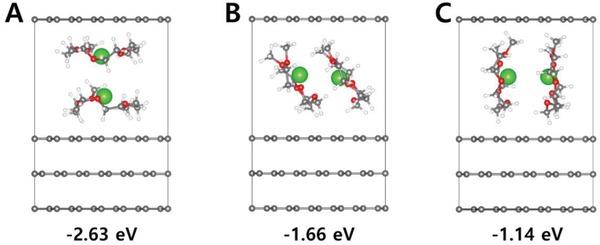
Ca‐G_4_·C_101_ (stage‐III) structures and corresponding intercalation energies for three different Ca‐G_4_ arrangements. Two aligned Ca‐G_4_ complexes were inserted into an interlayer of graphenes with A) parallel, B) oblique, and C) perpendicular orientations to graphene layers.

Based on the Ca‐G_4_ structure optimized by the Gaussian DFT calculation, a plausible arrangement of solvated Ca‐G_4_ within a graphene interlayer gap was estimated via DFT calculation using the plain wave basis set. We selected a stage‐III compound with a composition of Ca‐G_4_·C_101_, which corresponded to a reversible capacity of 44 mAh g^−1^ at −2.60 V. Based on our experimental results, we constructed a supercell 6 × 6 × 1.5 (A/B/A/2Ca‐G_4_/A stacking sequence along the c‐axis). In particular, double‐stacked Ca‐G_4_ complexes with three different orientations (parallel, oblique, and perpendicular to graphene layers) were incorporated into an interlayer gap of 11.41 Å (**Figure** **4**). A parallel insertion proved to be the most energetically favorable in terms of intercalation energy. The intercalation energy was evaluated using the total energy of Ca‐G_4_·C_101_ (*E*_Ca‐G4‐C_) and the sum of the energies of a Ca‐G_4_ complex molecule (*E*_Ca‐G4_) and pristine graphene (*E*_g_), as shown in Equation (4) below

(4) $$\mathrm{Intercalation}\;\mathrm{energy}=E_{\mathrm{Ca‐G}4\mathrm{‐C}}-\left(E_{\mathrm{Ca‐G}4}+E_{g}\right)$$

**Figure 5:**
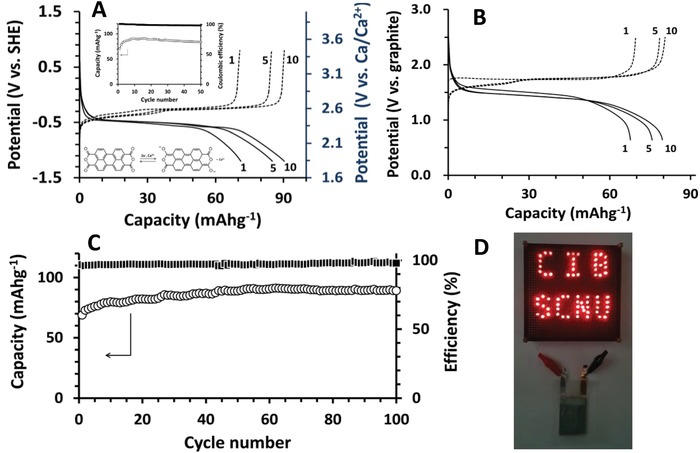
A) Representative C/D profiles (1st, 5th, and 10th) of a PTCDA half cell at 0.05 A g^−1^. Insets (top): Capacity retention and Coulombic efficiency; (bottom): 2‐electron redox process of PTCDA. B) Representative C/D profiles (1st, 5th, and 10th, The vertical axis indicates the voltage of PTCDA with respect to the voltage of graphite). C) Capacity retention and Coulombic efficiency of a full cell (Ca^2+^‐loaded graphite│Ca(TFSI)_2_/G_4_│PTCDA) at 0.05 A g^−1^. D) Photo of a LED lettering panel lit by a pouch‐type full cell.

Finally, we implemented graphite in a full cell to realize Ca^2+^‐shuttling CIBs. Prior to the use of perylene‐3,4,9,10‐tetracarboxylic dianhydride (PTCDA) as a cathode in a full cell, the Ca^2+^‐storage capability of PTCDA was examined in a half cell configuration with an AC counter electrode. As in monovalent ion batteries (Li^+^, Na^+^, and K^+^),^58^, ^59^, ^60^, ^61^, ^62^ PTCDA was also electroactive in Ca(TFSI)_2_/G_4_. PTCDA showed a gradual increase in capacities during the initial 10 cycles, which was followed by stable electroactivity in Ca(TFSI)_2_/G_4_ (**Figure** **5**A). The reversible capacities of ≈90 mAh g^−1^ (theoretical capacity = 137 mAh g^−1^, based on a 2‐electron process) were relatively high when a strong solvation in Ca‐G_4_ was considered.

PTCDA was also electroactive in carbonates containing Ca(TFSI)_2_ (Figure S11, Supporting Information). The C/D profiles and capacity values in carbonates were similar to those in G_4_, which suggested the possible involvement of bare Ca^2+^ for charge neutrality of PTCDA during C/D. The cyclic stability was as good as maintaining 96% of the initial capacity after 50 cycles at 0.05 A g^−1^.

For a full cell construction, Ca^2+^‐loaded graphite electrodes were electrochemically prepared and coupled with PTCDA. Based on the relative capacities, a 10% excess amount of Ca^2+^‐loaded graphite was implemented. Galvanostatic C/D was performed at 0.05 A g^−1^, and the cell was discharged first (Figure 5B). The 1st discharge capacity of 71 mAh g^−1^ was increased to ≈82 mAh g^−1^ during initial cycles (capacities based on a PTCDA mass), which was followed by a continuous increase to ≈90 mAh g^−1^ during 50 cycles (Figure 5C). Thereafter, a stable capacity of ≈90 mAh g^−1^ was maintained until the end of 100 cycles, with no variation in C/D potentials, which indicated that a graphite anode is a sustainable electrode even in a full cell configuration and graphite‐implemented CIBs can be made more viable by discovering cathode materials with higher performance.

The rate performance of a full cell was also examined (Figure S12, Supporting Information). A stepwise decrease in capacities was observed with an increase in current densities from 25 to 200 mA g^−1^, mostly reflecting the rate capability of PTCDA. For example, a decrease of discharge capacity to ≈47 mAh g^−1^ at 100 mA g^−1^ was believed due to the low rate‐performance of PTCDA, because graphite showed an excellent level of capacities at high rates (Figure 1H). The capacity was promptly restored to the initial capacity of ≈86 mAh g^−1^ when the current density was returned to 25 mA g^−1^. Finally, a pouch‐type full cell was fabricated, maintaining the same mass ratio between two electrodes. Figure 5D shows a photo of the LED lettering panel lit by a pouch‐type full cell during discharge. 69 LED lamps (11.6 mW) could be continuously lit for ≈20 min. Figure 5, therefore, conceptually prove that graphite can be successfully implemented as an anode in a rocking‐chair type CIB full cell.

## 3 Conclusion

We demonstrated that graphite can reversibly accommodate G_4_‐solvated Ca^2+^. When charged (reduced), graphite formed a ternary intercalation compound, Ca‐G_4_·C_72_, with no calcium plating. Ca‐G_4_ was reversibly intercalated/deintercalated during subsequent cycles, which resulted in a reversible capacity of 62 mAh g^−1^ at 0.05 A g^−1^. Excellent rate capability and cyclic stability were also achieved. Despite a large volume change during C/D, graphite delivered a reversible capacity of 47 mAh g^−1^ at a fast C/D rate of 1.0 A g^−1^ and showed no capacity decay during 2000 C/D cycles. The excellent electrochemical performance of graphite in Ca(TFSI)_2_/G_4_ motivated us to construct a full cell coupled with a PTCDA cathode. The rocking‐chair type CIB showed a reversible capacity >80 mAh g^−1^ and good stability for 100 C/D cycles with a reversible potential of ≈1.6 V versus graphite and Coulombic efficiency >98%. We hope that the results presented here will draw more attention to CIBs and provoke new ideas among CIB researchers.

## 4 Experimental Section

*Materials and Electrochemical Measurements*: Cu foil (25 µm, Wellcos) was cleaned with ethanol and dried at 60 °C under vacuum before use. Graphite powders, KS6L (TIMCAL, C‐NERGY, USA) and MCMB (MTI, USA), Ca(TFSI)_2_ (Solvionic, France), and PVFc (Polysciences Inc., USA) were used without further purification. Polyvinylidene fluoride (PVdF) binder (MTI, USA), conductive acetylene black (AB, MTI, USA), and HOPG electrode (Park systems, CA, USA) were used as received. Carbon cloth (CC, 356 µm, Fuel Cells, USA) was rinsed and sonicated in absolute ethanol for 2 min and dried under vacuum at 100 °C for 1 h before use. All the other materials were purchased from Sigma‐Aldrich, unless otherwise mentioned. Ca(TFSI)_2_ was dried at 100 °C for 3 h under vacuum before use and the solvents were used as received without further purification. Unlike Ca(TFSI)_2_, the other commercially available salts (Ca(BF_4_)_2_ and Ca(BH_4_)_2_ from Aldrich) were sparingly soluble in glyme‐type solvents and were not utilized for this study. For the preparation of glyme‐based electrolytes, Ca(TFSI)_2_ was dissolved in G_1_, G_2_, G_3_, and G_4_ to be 1.0 m. While the colorless solutions were readily made in G_2_ and G_4_, the complete dissolution of Ca(TFSI)_2_ in G_1_ and G_3_ required extensive stirring. For comparative studies, carbonate‐based electrolytes were also prepared. An appropriate amount of Ca(TFSI)_2_ was dissolved in PC and mixed carbonate solvents of EC/DEC (PuriEl, Korea) and EC:DMC:EMC. The latter was made by mixing the commercial EC/DMC (PuriEl, Korea) and EMC (Aldrich).

The graphite electrode was a mixture of 90 wt% graphite, 5 wt% PVdF, and 5 wt% AB. The PTCDA electrode was made by mixing 50 wt% PTCDA (Sigma‐Aldrich), 35 wt% PVdF, and 15 wt% AB. The slurry was coated onto Cu foil by a doctor blade method, which was then punched into the required size after drying and roll pressing. For the AC electrodes, a cleaned CC was punched into the desired size and an AC slurry (80 wt% AC, 10 wt% PVdF, and 10 wt% AB) was coated evenly on the CC electrodes, which was followed by drying at 120 °C for 30 min, and then by vacuum‐drying at 100 °C for 1 h. The AC coated CC electrode was used as a counter/reference electrode throughout the experiments. To minimize the potential variation (± 0.05 V) in an AC electrode during C/D, excess amount of AC was used relative to graphite. The coin cells (CR2032, Wellcos) were fabricated in an Ar‐filled glove box (O_2_, H_2_O < 1 ppm). Cyclic voltammetry measurements were performed using a Swagelok type cell. Pouch‐type full cells were fabricated by assembling a graphite anode and a PTCDA cathode with polyethylene terephthalate films. C/D cycle tests were performed using an automatic WBCS 3000 battery cycler (WonATech). The electrochemical impedance spectrum was recorded by applying a sine wave with amplitude of 10.0 mV at frequencies ranging from 100 kHz to 0.01 Hz.

For visual observation of the volume expansion of graphite due to cointercalation of Ca^2+^ and G_4_, HOPG (dimension of 0.5 × 1.0 × 0.12 cm) was pasted onto a Pt foil using a carbon paste. A long strip of CC was coated with an excessive amount of AC (23‐fold heavier than graphite) and used as a counter/reference electrode. Two electrodes facing each other were fixed inside a rectangular glass cell. The volume change was observed by a digital camera, which was positioned perpendiclar to the plane of HOPG. Precise measurements of HOPG volume change were made three times, which were subsequently subjected to digital analysis. For the observation of mass change of graphite due to cointercalation of Ca^2+^ and G_4_, weight changes of the graphite electrodes were checked at different state of charge after disassembling the coin cells. To exclude weight changes caused by residual solvent, the electrodes were dried at 60 °C for 12 h.

*Characterizations*: Ex situ XRD patterns were recorded using a Rigaku ULTIMA 4 equipped with Cu K_α_ radiation at a scan rate of 2° min^−1^. The electrode was protected in an air‐tight sample holder during analysis. In‐operando XRD patterns for graphite were collected on the 9A XRD beamline at PLS‐II, Postech, Pohang, South Korea. The wavelength of the X‐ray beam was 0.7653 Å, and XRD patterns were recorded as a set of circles on a Mar 345‐image plate detector in transmission mode for ≈7 s. For the in operando measurements, a coin‐type half cell (graphite│1.0 m Ca(TFSI)_2_/G_4_│AC) with a pinhole was constructed and C/D cycled at a current density of 0.05 A g^−1^. XPS studies were carried out using a Thermo Fisher (K‐Alpha) electron spectrometer with an Al K_α_ X‐ray source (excitation energy = 1486.6 eV). The peak positions were calibrated using C_1s_ (C−C, 284.8 eV), since it was believed that C−C (or C=C) is the dominant carbonic species in graphite. Field emission scanning electron microscopy investigations were performed using a JEOL JSM‐7100F equipped with an EDX spectroscope. For the XPS and EDX mesurements, the sampling was carried out inside an Ar‐filled glovebox and the sample stage was transferred to the instrument in a custom built transfer chamber, which ensured no exposure to air. Synchrotron‐based XPS was performed at the 10A2 HR‐PES II beamline, Postech, Pohang, South Korea, which can cover the photon energy of 100 (124 nm)–1600 (0.775 nm) eV with good photon resolution (*E*/Δ*E* = ≈5000). Raman spectra were recorded by a Jasco NRS‐2100 Laser Raman Spectrometer using a 532 nm laser line.

*DFT Calculations*: Relaxation of the molecular structure of a solvated Ca^2+^ ion was performed using ab initio calculations at the B3LYP/6‐31G* level based on a Gaussian 09 program (Gaussian, Inc., Wallingford, CT, USA). The solvation energy was calculated through counterpoise correction in order to avoid BSSE (basis set superposition error). The structural relaxation for a stage‐III ternary intercalation compound was performed using a VASP (Vienna Ab Initio Simulation Package). The PAW (projector augmented wave) pseudopotential^63^, ^64^ and PBE (Perdew–Burke–Ernzerhof)^65^ parameterized GGA (generalized gradient approximation) were adopted, and the van der Waals interaction was taken into account using DFT‐D3 potential.^66^

## Conflict of Interest

The authors declare no conflict of interest.

## Supporting information

Supplementary

Supplementary
