# Supplementary material for: Graphite as a Long‐Life Ca2+‐Intercalation Anode and its Implementation for Rocking‐Chair Type Calcium‐Ion Batteries
Source: Adv Sci (Weinh). 2019 Oct 16;6(24):1902129. doi: 10.1002/advs.201902129 (PMC6918123; doi:10.1002/advs.201902129)
Supplement: Supplementary file 1 — Supplementary [file ADVS-6-1902129-s001.pdf]

Copyright WILEY-VCH Verlag GmbH & Co. KGaA, 69469 Weinheim, Germany, 2019.

ADVANCED SCIENCE
Open Access

Supporting Information

for *Adv. Sci.,* DOI: 10.1002/advs.201902129

Graphite as a Long-Life Ca^{2+}-Intercalation Anode and its Implementation for Rocking-Chair Type Calcium-Ion Batteries

S. J. Richard Prabakar, Amol Bhairuba Ikhe, Woon Bae Park, Kee-Choo Chung, Hwangseo Park, Ki-Jeong Kim, Docheon Ahn, Joon Seop Kwak, Kee-Sun Sohn, and Myoungho Pyo**

Copyright WILEY-VCH Verlag GmbH & Co. KGaA, 69469 Weinheim, Germany, 2019.

Supporting Information

Graphite as a Long-Life Ca^{2+}-Intercalation Anode and its Implementation for Rocking-Chair Type Calcium-Ion Batteries

S. J. Richard Prabakar, Amol Bhairuba Ikhe, Woon Bae Park, Kee-Choo Chung, Hwangseo Park, Ki-Jeong Kim, Docheon Ahn, Joon Seop Kwak , Kee-Sun Sohn and Myoungho Pyo**

Dr. S. J. R. Prabakar, A. B. Ikhe, Prof. J. S. Kwak, Prof. M. Pyo
Department of Printed Electronics Engineering, Sunchon National University,
Chonnam 57922, Republic of Korea.
E-mail: mho@sunchon.ac.kr

Dr. W. B. Park, Prof. K-S. Sohn
Faculty of Nanotechnology and Advanced Materials Engineering, Sejong University,
Seoul 05006, Republic of Korea.
E-mail: kssohn@sejong.ac.kr

K-C. Chung, H. Park
Department of Bioscience and Biotechnology, Sejong University, 209 Neungdong-ro,
Kwangjin-gu, Seoul 143-747, Republic of Korea.

K-J. Kim, Dr. D. Ahn
Beamline Division, Pohang Accelerator Laboratory (PAL), Pohang 37673, Republic of Korea.

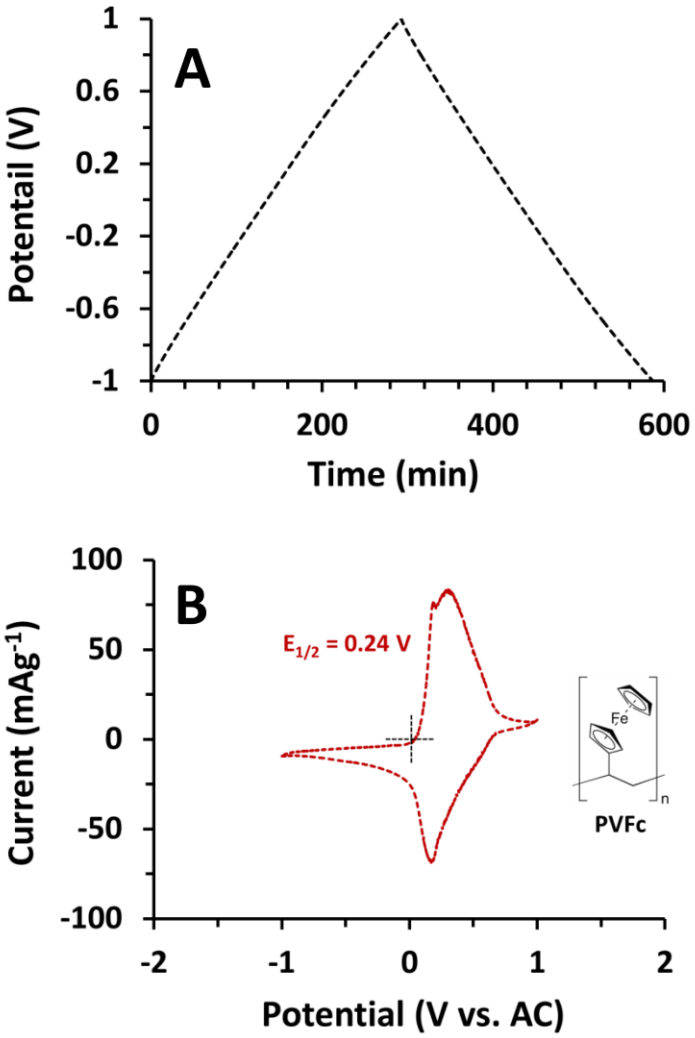

Figure S1. (A) C/D profile of a symmetric AC/AC cell at a current density of 20 mA·g^{-1} and (B) cyclic voltammogram of PVFc at a scan rate of 0.5 mV·s^{-1} in 1.0 M Ca(TFSI)$_2$/tetraglyme vs. AC. Capacitance of AC was determined to be 150 F·g^{-1} after subtracting the contribution of a substrate (carbon cloth) to the charge storage.

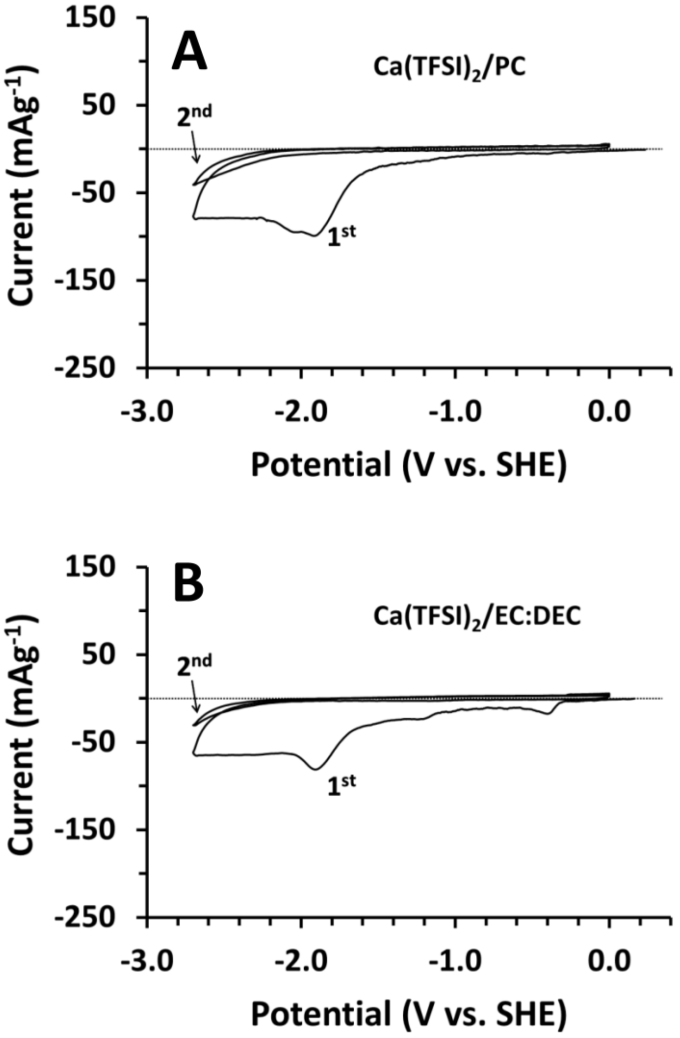

Figure S2. Cyclic voltammograms of graphite in (A) propylene carbonate (PC) and (B) ethylene carbonate:diethyl carbonate (EC:DEC, 5:5) containing 1.0 M $Ca(TFSI)_2$. Scan rate = 0.5 $mV{\cdot}s^{-1}$. No electroacitivity during the 2nd cycle results from the surface film, which is formed by electrolyte decomposition during the 1st negative scan.

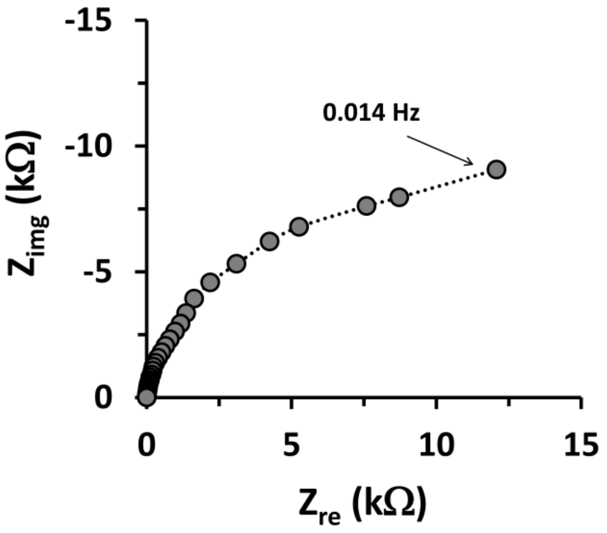

Figure S3. Electrochemical impedance spectra of graphite in $Ca(TFSI)_2$/EC:DMC:EMC (4:3:2) after charge (-2.7 V). The significant impedance after charge is obvious due to the surface passivation.

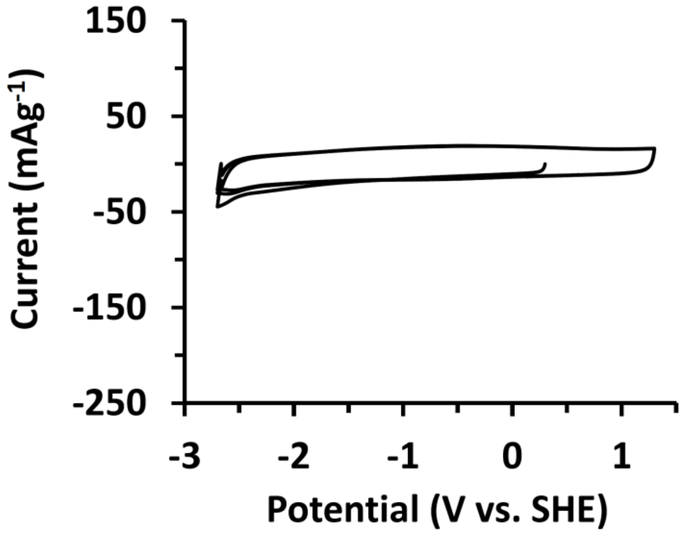

Figure S4. Cyclic voltammograms of AC in $Ca(TFSI)_2/G_4$ at 0.5 $mV\cdot s^{-1}$. When compared with votammograms in Figure 1B ~ 1E, the magnitude of currents is incomparably low and no characteristic features for a Faradaic process are seen.

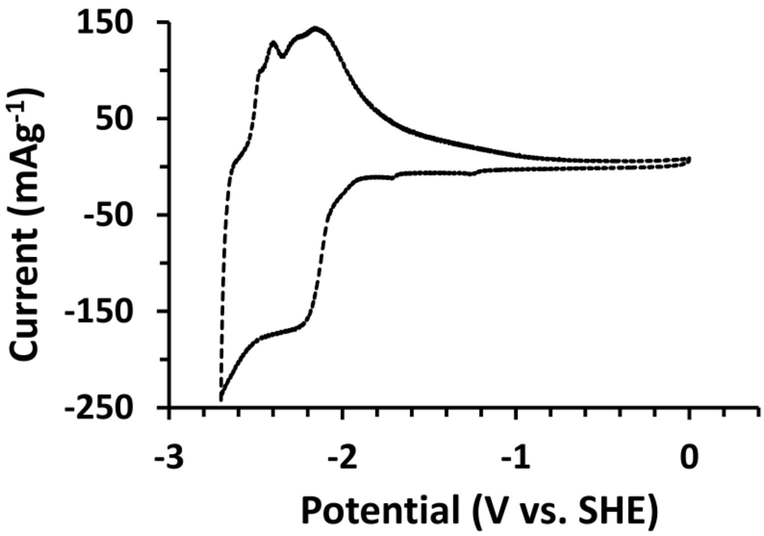

Figure S5. Cyclic voltammogram of MCMB graphite at a scan rate of 0.5 mV·s^{-1} in 1.0 M $Ca(TFSI)_2/G_4$, which shows behaviors similar to that in Figure 1E.

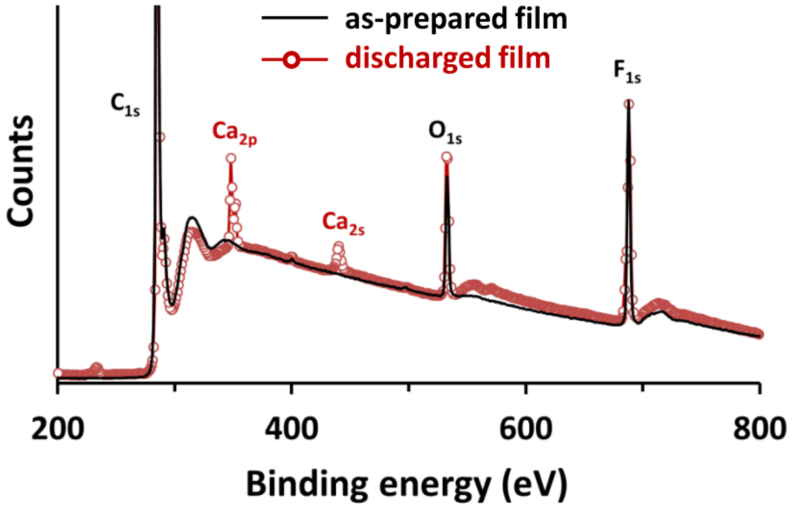

Figure S6. Survey XPS spectra of as-made and fully discharged films (-0.2V vs. SHE). For the latter, graphite film was cycled in $Ca(TFSI)_2/G_2$. A film in a discharged state shows a high concentration of calcium, which implies Ca^{2+} entrapment. The identical intensities of 'F' from PVdF binder suggest that the high concentration of calcium does not result from a solid-electrolyte-interface layer, but from Ca^{2+} ions entrapped within graphite.

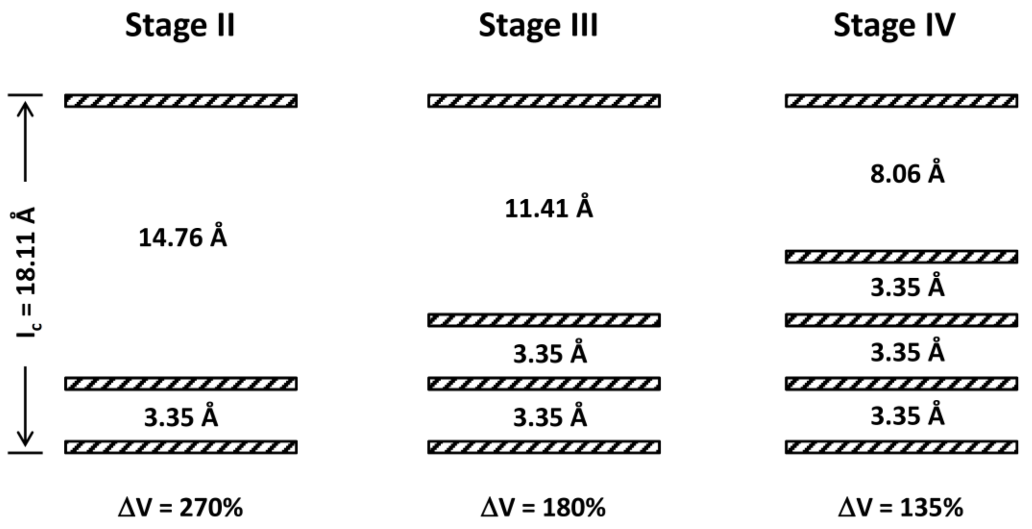

Figure S7. Possible stages of graphite-intercalation-compounds with I_c = 18.11 Å. The formation of the stage II and stage IV was not plausible because of ΔV values that are inconsistent with the HOPG expansion in Figure 3A.

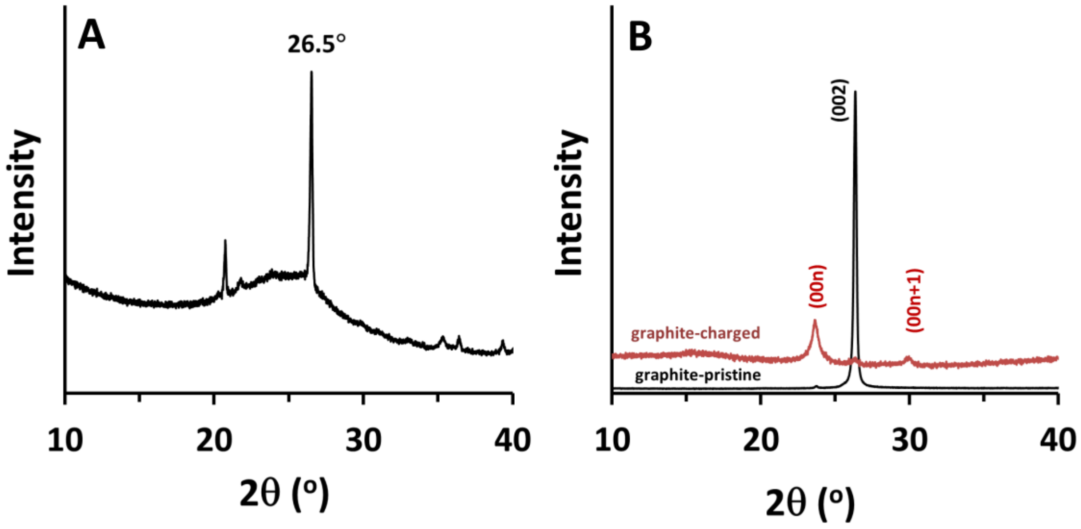

Figure S8. (A) XRD pattern of AC. (B) Ex-situ XRD patterns of pristine graphite and fully charged graphite after 3 C/D cycles. The intensity of pristine graphite was reduced by 60 % for clarity. The charged graphite shows almost complete suppression of the (002) peak, which indicates that the strong (002) peak shown in the in-situ XRD patterns is due to the AC counter electrode.

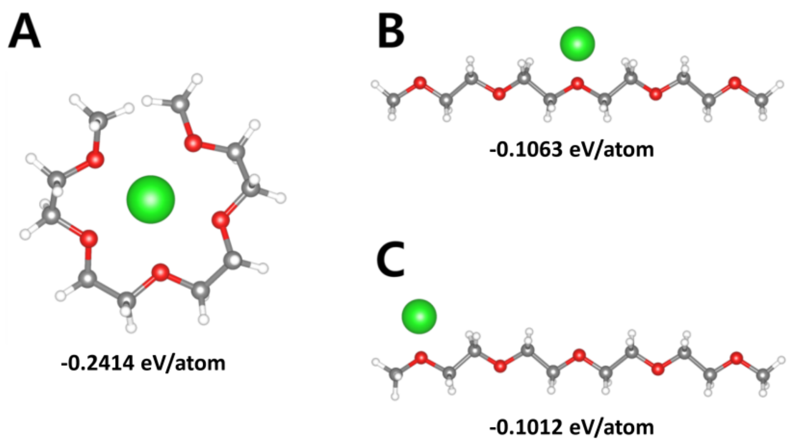

Figure S9. Comparison in DFT-calculated solvation energies of fully optimized Ca-G_4 complexes with (A) circular and (B & C) straight G_4 molecules. Ca^{2+}, C, O, and H are presented as green, red, grey, and white balls, respectively.

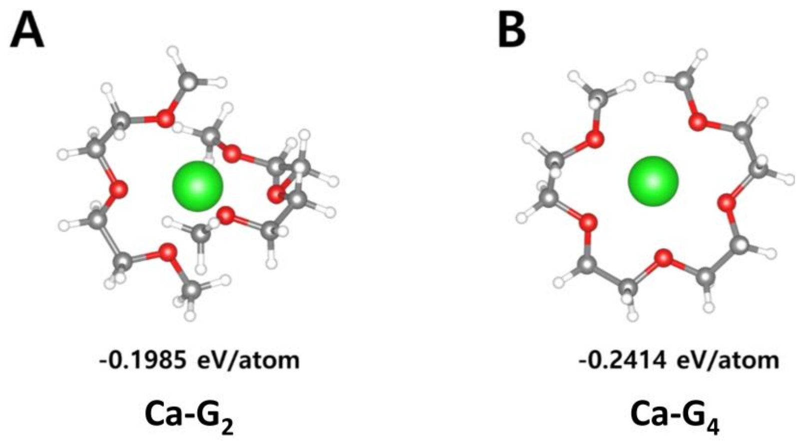

Figure S10. The DFT-optimized structure of (A) Ca-G_2 and (B) Ca-G_4 complexes with their corresponding solvation energies. Ca^{2+}, C, O, and H are presented as green, red, grey, and white balls, respectively.

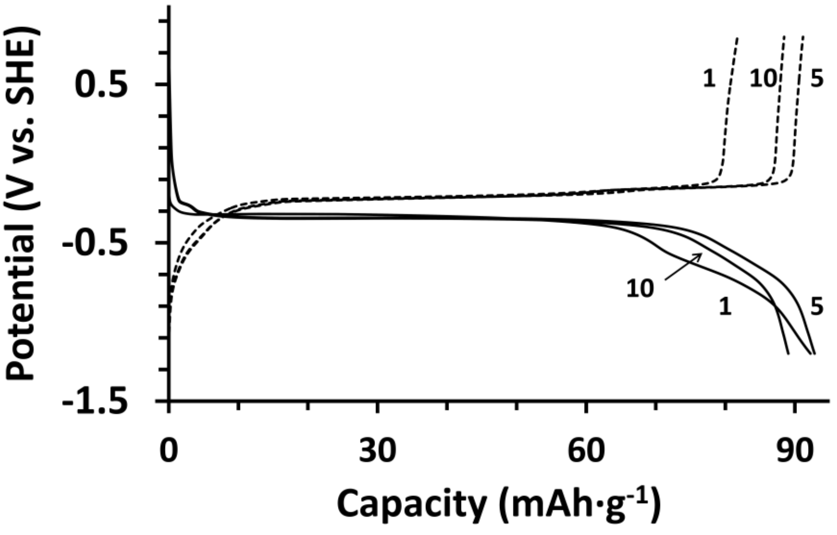

Figure S11. C/D profiles of PTCDA (half cell) in ethylene carbonate:diethyl carbonate (EC:DEC, 5:5) containing 1.0 M $Ca(TFSI)_2$ at 0.05 $A{\cdot}g^{-1}$.

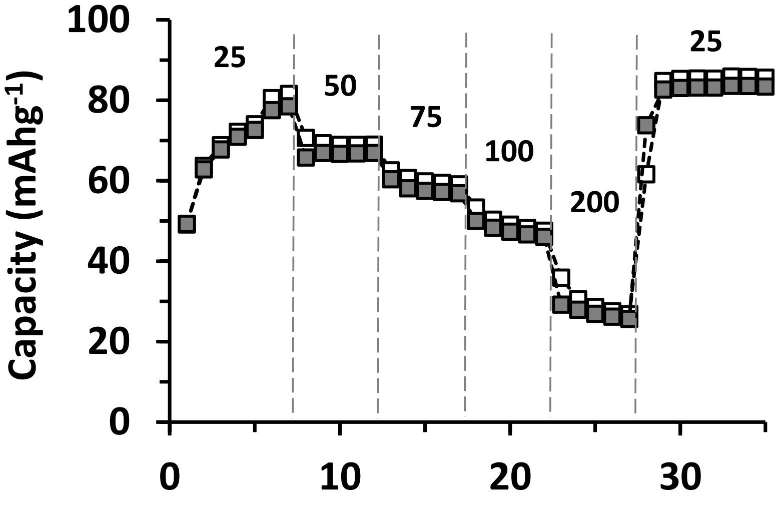

Figure S12. Rate performance of a full cell (Ca^{2+}-loaded graphite|$Ca(TFSI)_2/G_4$|PTCDA). Numbers indicate the current density in $mA{\cdot}g^{-1}$. Solid mark: charge (Ca^{2+} extraction) capacity. Open mark: discharge (Ca^{2+} insertion) capacity.
